# Quantitative proteomic analysis of circulating exosomes reveals the mechanism by which Triptolide protects against collagen‐induced arthritis

**DOI:** 10.1002/iid3.1322

**Published:** 2024-06-18

**Authors:** Xiuchan Liu, Xu Liu, Hui Wang, Ming Chen, Geng Zhang, Dongyun Ren, Na Zhang, Wei Wei

**Affiliations:** ^1^ Department of Infectious Diseases Tianjin Hospital Tianjin China; ^2^ Department of Rheumatology and Immunology Tianjin Medical University General Hospital Tianjin China; ^3^ Department of Infectious Diseases Tianjin Medical University General Hospital, Binhai Hospital Tianjin China; ^4^ Department of Infectious Diseases Tianjin Medical University Baodi Clinical College Tianjin China

**Keywords:** exosomes, inflammation, proteomics, rheumatoid arthritis, triptolide

## Abstract

**Introduction:**

Triptolide (TP), a natural product derived from the herbal medicine *Tripterygium wilfordii*, exhibits potent immunosuppressive activity. However, the mechanisms underlying its effects in rheumatoid arthritis remain incompletely understood.

**Methods:**

Collagen‐induced arthritis (CIA) model was induced in Sprague−Dawley rats by immunization with bovine type II collagen, and TP was administrated as treatment. The therapeutic effect of TP was evaluated based on paw swelling, histopathology, and serum levels of inflammatory factors. Exosomes isolated from rat serum were characterized by transmission electron microscopy, dynamic light scattering, and western blot analysis. Proteomic profiling of exosomes was analyzed by direct DIA quantitative proteomics analysis. Gene ontology and the Kyoto Encyclopedia of Genes and Genomes databases were employed for enrichment analysis related to molecular function, biological processes, and signaling pathways. Western blot analysis was used to analyze differentially expressed proteins.

**Results:**

TP treatment ameliorated arthritic phenotypes in CIA rats as evidenced by reduced arthritis score, paw swelling, pathological injury severity scores, and serum levels of inflammatory cytokines. The proteomic analysis revealed that TP treatment significantly inhibited complement and coagulation cascades, interleukin‐17 signaling pathway, and cholesterol metabolism, which were reactivated in CIA rats. Importantly, lipocalin 2 (LCN2) and myeloperoxidase (MPO) levels were markedly upregulated in the CIA group but suppressed upon TP administration. Furthermore, in synovial tissues, LCN2 and MPO expression levels were also elevated in the CIA group but decreased following TP treatment.

**Conclusion:**

Our findings demonstrate that TP alleviates CIA, possibly through modulation of exosomal LCN2 and MPO proteins.

## Graphical abstract

Triptolide (TP) treatment resulted in changes in many circulating exosomal proteins in collagen‐induced arthritis (CIA) rats. These proteins were enriched in complement and coagulation cascades, IL‐17 signaling pathways, and cholesterol metabolism‐related proteins. TP alleviated CIA, which may be associated with the modulation of exosomal lipocalin 2 and myeloperoxidase proteins.

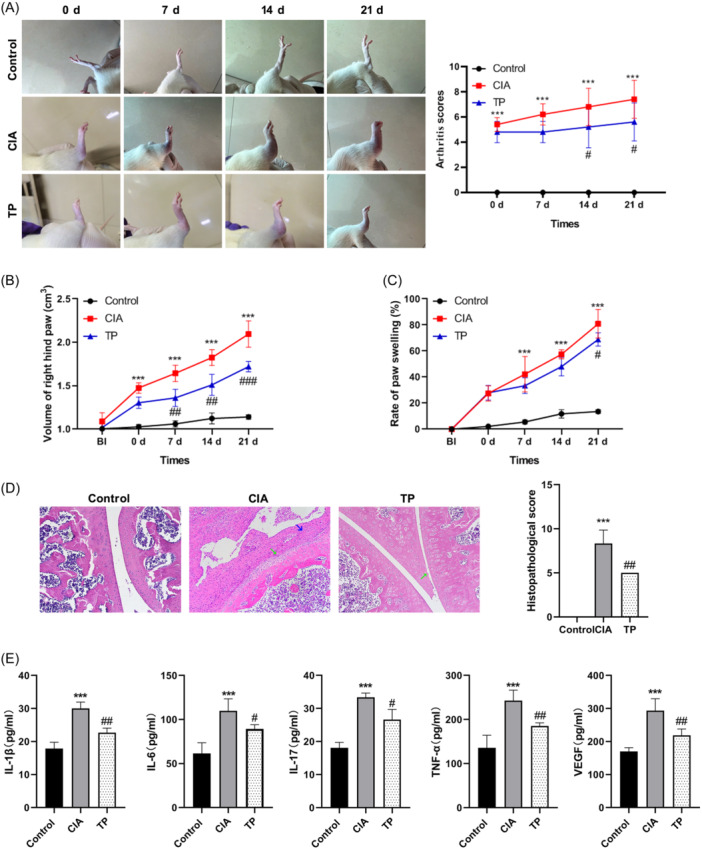

## 1 INTRODUCTION

Rheumatoid arthritis (RA) is a prevalent autoimmune disease characterized by symmetrical polyarthritis, progressive cartilage destruction, and bone erosion. The global prevalence is estimated to range from 0.5% to 1.0%, with a higher incidence in women than men.^1^ Although the pathological mechanism of RA is not clearly unclear, it may involve genetic and environmental factors, gene‐environment interactions, and epigenetic modification.^2^ In current clinical practice, nonsteroidal anti‐inflammatory drugs, antirheumatic drugs, glucocorticoids, and biological agents are commonly used to control the progression of RA. While these drugs can effectively inhibit inflammation and alleviate RA symptoms in the short term, their long‐term application may lead to gastrointestinal reactions, renal insufficiency, heart diseases, and other side effects.^3^ Therefore, effective prevention or intervention strategies for preventing the progression of RA have become important clinical concerns. Consequently, searching for novel approaches or drugs for managing RA patients has gained significant importance.

*Tripterygium wilfordii* Hook F (TwHF), a traditional Chinese herb widely used in various autoimmune diseases and tumors due to its potent anti‐inflammatory and immunosuppressive activities, holds promise as a potential treatment option.^4^, ^5^ Triptolide (TP), a natural bioactive product isolated from TwHF root extract, exhibits antiarthritic, anti‐inflammatory, antitumor, and neuroprotective properties. TP has been reported to reduce pain associated with RA while improving morbidity outcomes.^6^, ^7^ However, further exploration is required regarding its mechanisms of action within the context of RA pathogenesis.

Exosomes are small extracellular vesicles ranging from 30 to 150 nm. As a noncontact intercellular communication, exosomes facilitate the exchange of materials and information between cells by transporting active proteins and nucleic acids. The content and function of exosomes are dependent on their secretory cells and microenvironment. Recently, the interaction between exosomes and natural bioactive products has attracted more and more attention. Exosomes possess characteristics such as secretion, specific targeting, biological functionality, and biocompatibility that align with the material basis requirements of natural compounds theory.^8^ These characteristics suggest that exosome‐mediated intercellular communication may serve as an important mechanism for natural compounds to exert pharmacological effects. Several studies have recently reported the role of exosomes in mediating the therapeutic effects of natural bioactive products in various diseases. For instance, Naringenin, a natural product known for its antioxidative stress properties, inhibits macrophage M1 polarization by reducing the release of exosomal miR‐21‐3p derived from BEAS‐2B cells.^9^ Schisandrol A, a bioactive constituent from Schisandrae Chinensis Fructus, has been reported to alleviate drug‐induced liver injury through autophagy activation via exosomes.^10^ Shikonin, a naphthoquinone isolated from Lithospermum, inhibits human breast cancer cell proliferation by reducing tumor‐derived exosomes.^11^ These findings indicate that natural bioactive products can modulate the secretion and function of exosomes to exert their pharmaceutical activities. Therefore, this study aims to investigate the mechanism underlying the effect of TP on RA based on the analysis of proteomics of exosomes.

Collagen‐induced arthritis (CIA) model is commonly used to study the pathogenesis and treatment potions for RA. This model involves immunizing animals with homologous or allogenic type II collagen (CII) to induce chronic polyarthritis. In this study, Sprague−Dawley (SD) rats were immunized with CII to establish the CIA models which were subsequently treated with TP. Exosomes isolated from these rats were subjected to quantitative proteomics analysis.

## 2 METHODS

### 2.1 Establishment of the CIA rat model and TP treatment

This study was approved by the Ethics Committee of Tianjin Hospital (No. 2023069). Male SD rats (7 weeks old, weighing 210 ± 14 g) were provided by the Charles River and maintained in a specific pathogen‐free room with a 12 h/12 h light/dark cycle. Rats were given standardized food and water ad libitum while being maintained at a temperature of (22 ± 2)°C and humidity of (50 ± 10)%. After adaptive feeding for 7 days, rats were used to induce the CIA model as previously described.^12^ Briefly, six rats were selected as controls, while 12 rats were immunized with bovine type II collagen (CII; Chondrex) emulsified in complete Freund's adjuvant (Chondrex). About 0.3 mL of CII emulsion (1.0 mg/mL) was subcutaneously injected at the tail base and back of each rat. After 1 week, the CII emulsion was given as the same preparation as booster immunization. The Control group received an equal volume of normal saline instead. Two weeks after primary immunization, CIA rats were randomly divided into two groups: model group (CIA group, *n* = 6) and TP group (*n* = 6). TP can markedly decrease arthritis severity in CIA rats when administered at a dose of 45 μg/kg as previously described^13^, ^14^; therefore, this dosage of TP (MCE, HY‐32735; Purity: 99.86%) was chosen for our experiment. The TP group was treated with 45 μg/kg/day of TP by gavage (MedChemExpress) for 21 days. The CIA group and Control group were given the same volume of distilled water.

### 2.2 Model evaluation

The paw volume was measured and the arthritis score was calculated before immunization (BI), 0, 7, 14, and 21 days after TP treatment, respectively. The paw volume of the right hind limb was measured with a small animal toe volume measuring instrument. Paw swelling rate = (rat paw volume−basal paw volume)/basal paw volume × 100%. Arthritis score was determined by the grade of 0−4: 0, no joint swelling; 1, mild swelling of the little toe joints; 2, moderate swelling of the toe joints and ankle; 3, obvious swelling of the paw; 4, severe swelling involving entire paw, including the ankle. The arthritis index is the cumulative score for all four paws.^15^

### 2.3 Hematoxylin and eosin (H&E) staining

After the experiment, the ankle joints of rats were dissected and rinsed with phosphate buffer saline Subsequently, they were fixed in 10% formalin solution, decalcified, dehydrated, transparent, waxed, embedded, and sliced. The paraffin sections underwent sequential immersion in xylene, high to low‐concentration alcohol solutions, and distilled water. Hematoxylin staining was performed for 5 min followed by rinsing. Ethanol hydrochloride differentiation was carried out for 30 s. After soaking in warm water for 5 min, the sections were placed in eosin dye solution for 2 min and then dehydrated, transparent. Finally, the sections were sealed and examined under the microscope. The severity of inflammatory cell infiltration, synovial hyperplasia, articular cartilage destruction, and cartilage and bone damage were assessed using a histopathological scoring system ranging from 0 to 3.^16^ Inflammatory infiltration: 0, no inflammatory infiltration; 1 point, slight infiltration; 2, moderate infiltration; 3, severe infiltration. Synovial hyperplasia: 0, no hyperplasia; 1, mild synovial hyperplasia; 2, moderate synovial hyperplasia; 3: severe synovial hyperplasia. Cartilage destruction: 0, no destruction; 1, mild cartilage destruction; 2, moderate cartilage destruction; 3, severe cartilage destruction. Bone damage: 0, no damage; 1, mild damage; 2, moderate damage; 3, severe damage.

### 2.4 Enzyme‐linked immunosorbent assay (ELISA)

After the experiment, the rats were anesthetized, and blood was collected from the abdominal aorta. The collected whole blood was placed in a coagulant tube for natural coagulation, followed by centrifugation at 3000*g* for 15 min. Subsequently, the upper serum (100 µL) was transferred to a new centrifuge tube for cytokine detection. The concentration of interleukin‐1β (IL‐1β), IL‐6, IL‐17A, tumor necrosis factor‐alpha (TNF‐α), and vascular endothelial growth factor (VEGF) in serum was detected using ELISA kits (Jiangsu Kete Biotechnology Co., Ltd.). The absorbance value at 450 nm of each sample was measured using an automatic enzyme labeling instrument (Jinan Zhongan Biotechnology Service Co., Ltd.).

### 2.5 Exosome isolation

The collected whole blood was placed in a coagulant tube for natural coagulation and then centrifuged at 3000*g* for 15 min. The upper serum was transferred to a new centrifuge tube, and serum Exosome Isolation reagent (System Biosciences) was added in a ratio of 4:1 (serum volume: exosome isolation reagent volume). The mixture was repeatedly reversed and thoroughly mixed before standing on ice for 30 min. Subsequently, the mixture was centrifuged at 1500*g* for 30 min. After discarding the supernatant, the serum exosomes were obtained by further centrifugation at 1500*g* for 5 min followed by removal of the supernatant.

### 2.6 Transmission electron microscope (TEM)

Prepared exosomes (10 μL) were dropped onto the copper net with supporting film and adsorbed naturally for 5−10 min. Excess liquid was removed using filter paper and gently dried. Then, 20 μL of 2% phosphotungstic acid solution was added to the copper grid and incubated for 3−5 min. Finally, images were observed and captured under a TEM.

### 2.7 Western blot analysis

Exosomes from rat serum were lysed in radioimmunoprecipitation buffer and then centrifuged at 10,000*g* for 15 min. The supernatant‐containing protein was transferred to a new tube for bicinchoninic acid quantification. Twenty microliters of protein samples were separated by sodium dodecyl sulfate denatured 10% polyacrylamide gel electrophoresis. Subsequently, the gel was fixed in a sandwich clip and placed in the transfer buffer at a constant flow of 200 mA for 1 h, to promote the transfer of proteins from the gel to the polyvinylidene fluoride membrane for imprinting formation. The imprinted film was then placed in the sealing liquid and shaken at room temperature for 30 min. The membrane was cut according to the position of protein blotting, placed into the primary antibody diluent, and shaken overnight at 4°C. After rinsing three times with Tris‐buffered saline solution containing 0.1% Tween 20 detergent (TBST) solution for 5 min, the membrane was incubated with corresponding secondary antibodies (horseradish peroxidase peroxidase‐labeled sheep anti‐rabbit or mouse IgG antibody) at room temperature for 1 h. After rinsing, the membrane was exposed to Super super‐enhanced chemiluminescence (ECL) Plus super sensitive luminescent solution for 2 min. Immediately, the film was placed in the exposure box, exposed under darkroom conditions, and then developed and fixed. The antibodies used in this study are as follows. CD9 (YT0782, 1:500), CD63 (YT5525, 1:500), A2M (YT5368, 1:500), CA1 (YT0572, 1:500), and CA2 (YT0573, 1:500) polyclonal antibodies were obtained from Immunoway Biotechnology Company. CD81 antibody (ab109201, 1:1000) was purchased from Abcam. Lipocalin 2 (LCN2 antibody) (DF6816, 1:500) was from Affinity Biosciences, and the myeloperoxidase (MPO) antibody (AF7494, 1:500) was from Beyotime. GAPDH antibody (60004‐1‐Ig, 1:10,000) was obtained from Proteintech.

### 2.8 Liquid chromatography‐mass spectrometry/mass spectrometry (LC‐MS/MS) analysis of exosomal protein

Isolated protein was prepared with a commercially available iST Sample Preparation kit (PreOmics) for denaturation, reduction, alkylation, tryptic digestion, and peptide cleanup. The eluted peptide was vacuum‐dried and stored at −80°C until further use. Before LC‐MS/MS, the peptide was redissolved in 0.1% formic acid solution. The UltiMate 3000 liquid chromatography system (Thermo Fisher Scientific) and timsTOF Pro mass spectrometer (Bruker Daltonics) was employed for the analysis. A total of 200 ng of peptide was separated by analytical column (25 cm × 75 μm i.d.) with a 60 min gradient starting at 4% buffer B followed by a stepwise increase to 28% in 25 min, 44% in 10 min, 90% in 10 min and stayed there for 7 min, then equilibrates at 4% for 8 min. The flow rate of the column was maintained at 500 nL/min with a temperature of 50°C.

Data‐independent acquisition (DIA) data was acquired in the dia‐parallel accumulation‐serial fragmentation (PASEF) mode. We defined 22 × 40 Th precursor isolation windows from m/z 349 to 1229. To adapt the MS1 cycle time, we set the repetitions to variable steps (2−5) in the 13‐scan dia‐PASEF scheme in our experiment. During PASEF scanning, the collision energy was ramped linearly as a function of the mobility from 59 eV (1/K0 = 1.6 Vs/cm)^2^ to 20 eV (1/K0 = 0.6 Vs/cm^2^).

### 2.9 Gene ontology (GO) and Kyoto encyclopedia of genes and genomes (KEGG) pathway enrichment analysis

Blast2GO version 5 was utilized to annotate the function. GOATOOLS was used for GO enrichment analysis. The KOBAS database (http://kobas.cbi.pku.edu.cn/) was used for pathway analysis.

### 2.10 Statistical analysis

The statistical analysis was conducted using SPSS version 23.0 (SPSS Inc.). Data were expressed as mean ± standard error of the mean (SEM). The arthritis index was assessed using the nonparametric Kruskal−Wallis test, while other data were analyzed using analysis of variance followed by a post hoc test. A significance level of *p* < .05 was considered statistically significant.

## 3 RESULTS

### 3.1 TP treatment alleviates CIA‐related symptoms

**Figure 1:**
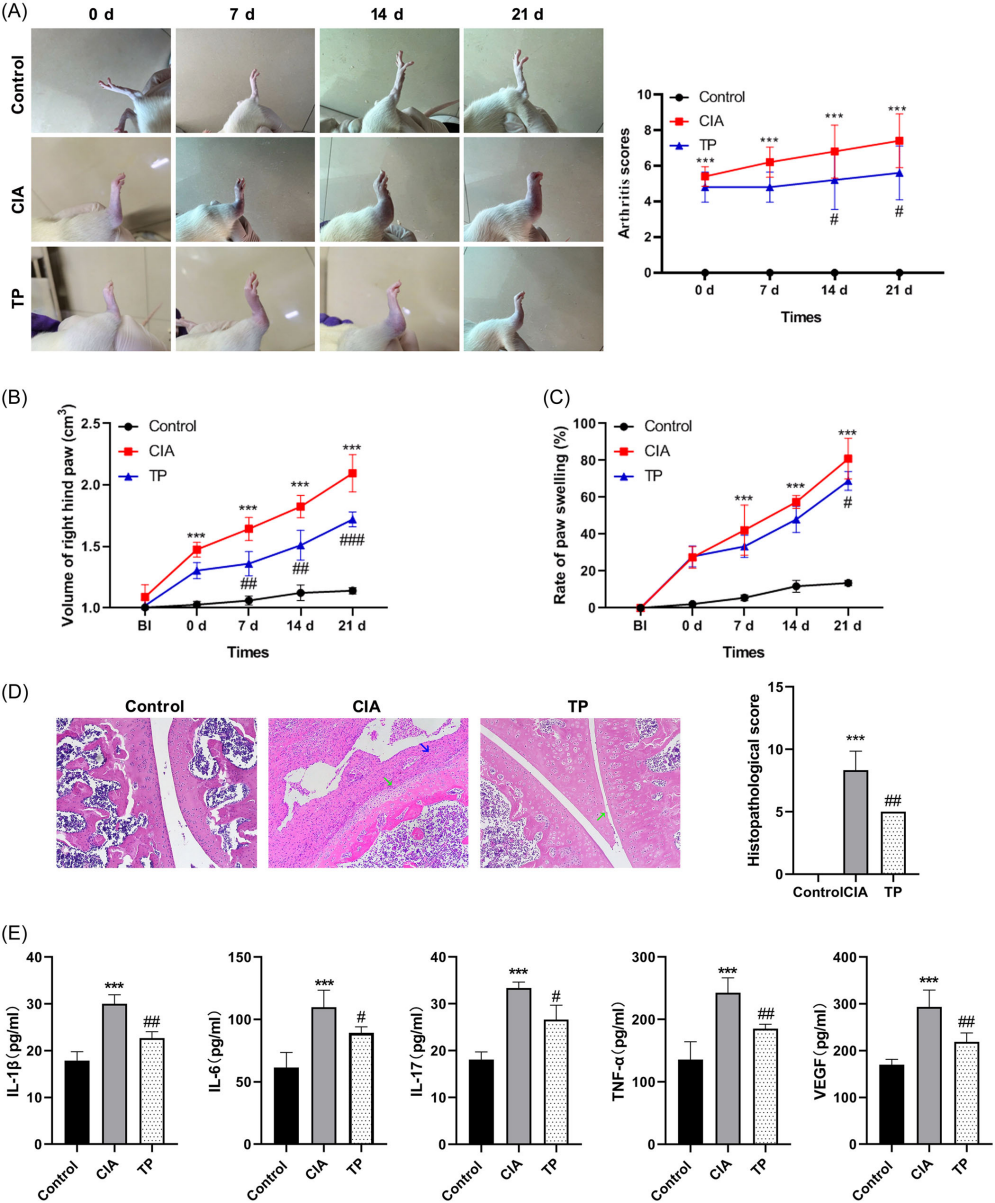
TP treatment alleviated collagen‐induced arthritis‐related symptoms. Rats were administered with TP (45 µg/kg) or vehicle for 21 days starting from the 15th day of initial immunization. (A) Morphological features of representative ankle joints (left). Arthritic scores were recorded on days 0, 7, 14, and 21 after TP treatment (*n* = 6). (B) Diameter of the right hind paw after TP treatment (*n* = 6). (C) Swelling rate of the right hind paw after TP treatment (*n* = 6). (D) Histopathological features of representative ankle joints (left), ×100,). Green arrows indicate cartilage destruction, and blue arrows indicate neutrophil infiltration. The right chart shows the histopathological score (*n* = 6). (E) ELISA assay was performed to determine the serum levels of IL‐1β, IL‐6, IL‐17A, TNF‐α, and VEGF in each group after TP treatment (*n* = 6). Data are presented as the mean ± SEM (*n* = 6). ***p < .001, versus the Control group, ^#^ *p* < .05, ^##^ *p* < .01, and ^###^ *p* < .001, versus the TP group. BI, before immunization; ELISA, enzyme‐linked immunosorbent assay; TNF, tumor necrosis factor‐alpha; TP, triptolide; VEGF, vascular endothelial growth factor.

SD rats were immunized with bovine CII for 2 weeks to establish a CIA model, followed by a 3‐week treatment with TP. Throughout the experiment, the rats in the Control group exhibited normal mental states, normal foraging and drinking habits, stable weight gain, smooth fur, and normal gait. CIA rats displayed poor mental state, loss of appetite, slow weight gain, inflexibility and even lameness, and dull hair on their back. However, compared to the model group, TP‐treated rats showed significant improvement in their overall condition. For arthritis‐related symptoms assessment, while the Control group demonstrated unrestricted movement patterns, the CIA model group exhibited decreased activity along with paw and ankle swelling. TP administration in the treatment group alleviated paw swelling and reduced arthritis scores from day 14th (Figure 1A). Furthermore, the TP‐treated group had lower paw volume and a slower rate of paw swelling compared to the CIA model group (Figure 1B,C).

Meanwhile, histopathological analysis revealed the damaged ankle joint structure and cartilage degradation, accompanied by inflammatory infiltration in CIA rats when compared to the Control group. Following TP treatment, these pathological characteristics improved significantly, and the histopathological score was notably decreased (Figure 1D). In addition, the ELISA results indicated significantly increased serum levels of IL‐1β, IL‐6, IL‐17A, TNF‐α, and VEGF in the CIA group when compared to the Control group. However, treatment with TP led to a significant reduction in these inflammatory cytokine levels (Figure 1E). These results collectively indicate that TP has therapeutic effects on alleviating joint destruction and suppressing inflammatory response in CIA rats.

### 3.2 Characterizations of exosomes

**Figure 2:**
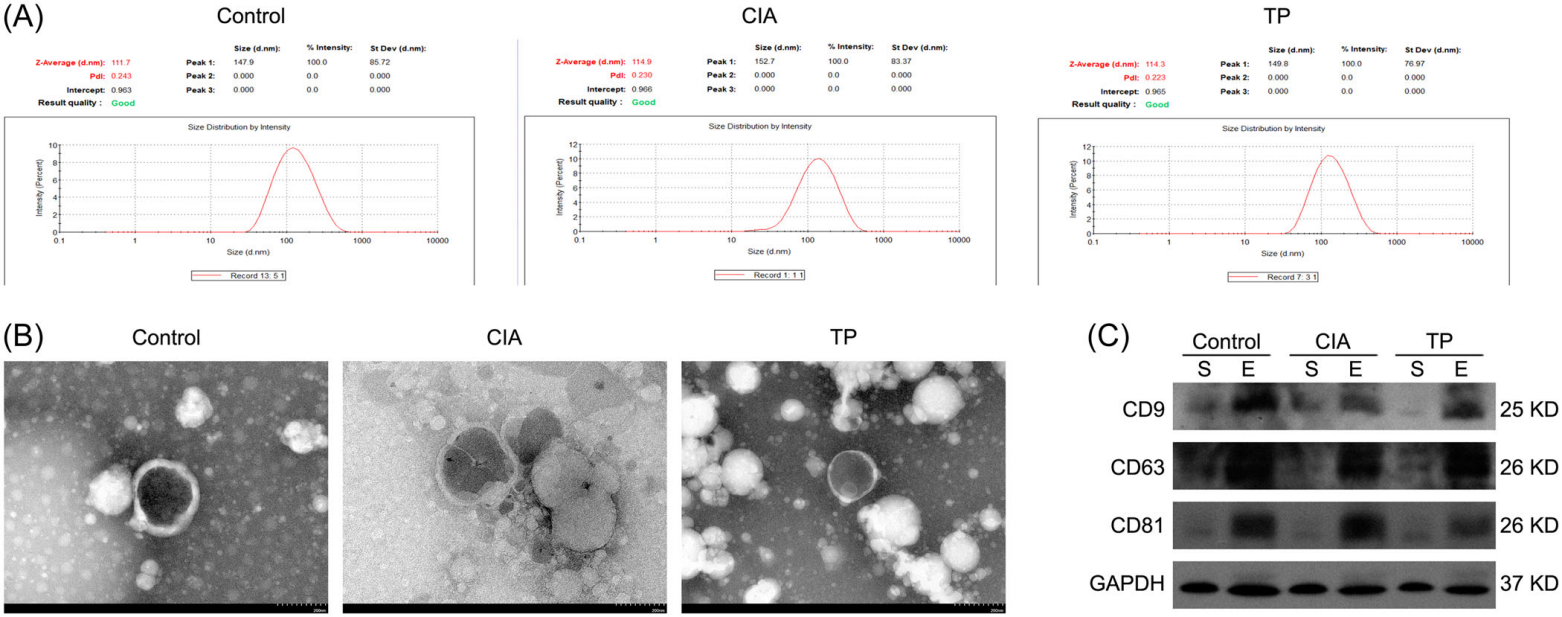
Characteristics of serum exosomes. (A) Particle size distribution of serum exosomes in each group, analyzed by nanoparticle tracking. (B) Transmission electron microscope images of exosomes. (C) Western blot analysis of CD9, CD63, and CD81.

Exosomes were isolated from rat serum using the ultracentrifugation method. The size distribution of exosomes ranged from 30 to 500 nm, as determined by dynamic light scattering (Figure 2A). Electron microscopy images confirmed the presence of exosomes with sizes around 150 nm (Figure 2B). Immunoblot analysis showed the positive expression of exosomal markers CD9, CD63, and CD81 (Figure 2C). These results confirmed the successful isolation of exosomes from rat serum.

### 3.3 Proteomics analysis in serum exosomes

**Figure 3:**
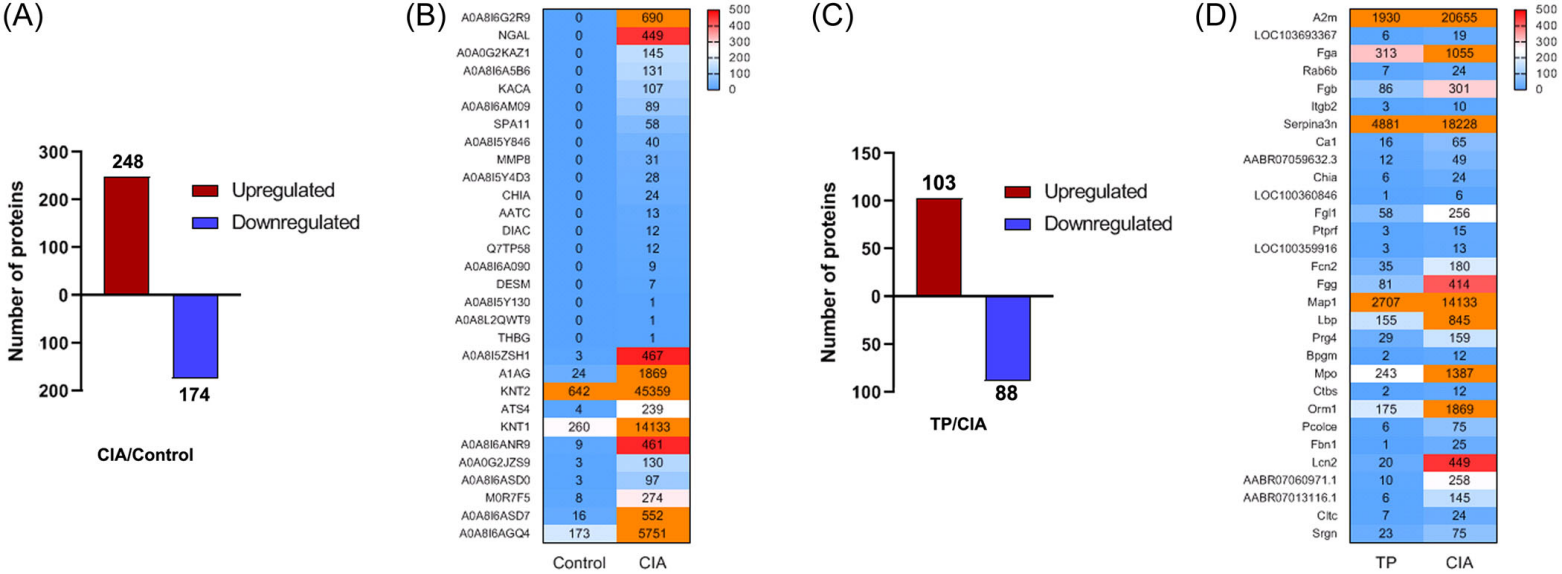
Analysis of differentially expressed proteins (DEPs) in serum exosomes. (A) Number of DEPs between the CIA and Control groups. (B) Heatmap depicting hierarchical clustering analysis of the top 30 highest expression levels of DEPs in the CIA group compared to the Control group. (C) Number of DEPs between the CIA and TP groups. (D) Heatmap depicting hierarchical clustering analysis of the top 30 lowest expressions of DEPs between the TP group compared to the CIA group. CIA, collagen‐induced arthritis; TP, triptolide.

The proteomics in serum exosomes was investigated to explore the impact of TP treatment on CIA mice using direct DIA‐based quantitative proteomics. A fold change (FC) threshold of ≥2.0 or ≤−2.0 was applied to identify differentially expressed proteins (DEPs). When comparing CIA to Control, we identified 422 DEPs, including 248 upregulated DEPs and 174 downregulated DEPs (Figure 3A). Heatmap analysis highlighted differences between the top 30 highest expressed known proteins in the CIA group (Figure 3B). Furthermore, a total of 191 DEPs was identified in TP‐treated rats compared to the CIA group, with 103 upregulated proteins and 88 downregulated proteins (Figure 3C). Hierarchical cluster analysis showed the top 30 lowest expressed proteins in the TP group compared to the CIA group (Figure 3D).

### 3.4 Function analysis of DEPs in serum exosomes

**Figure 4:**
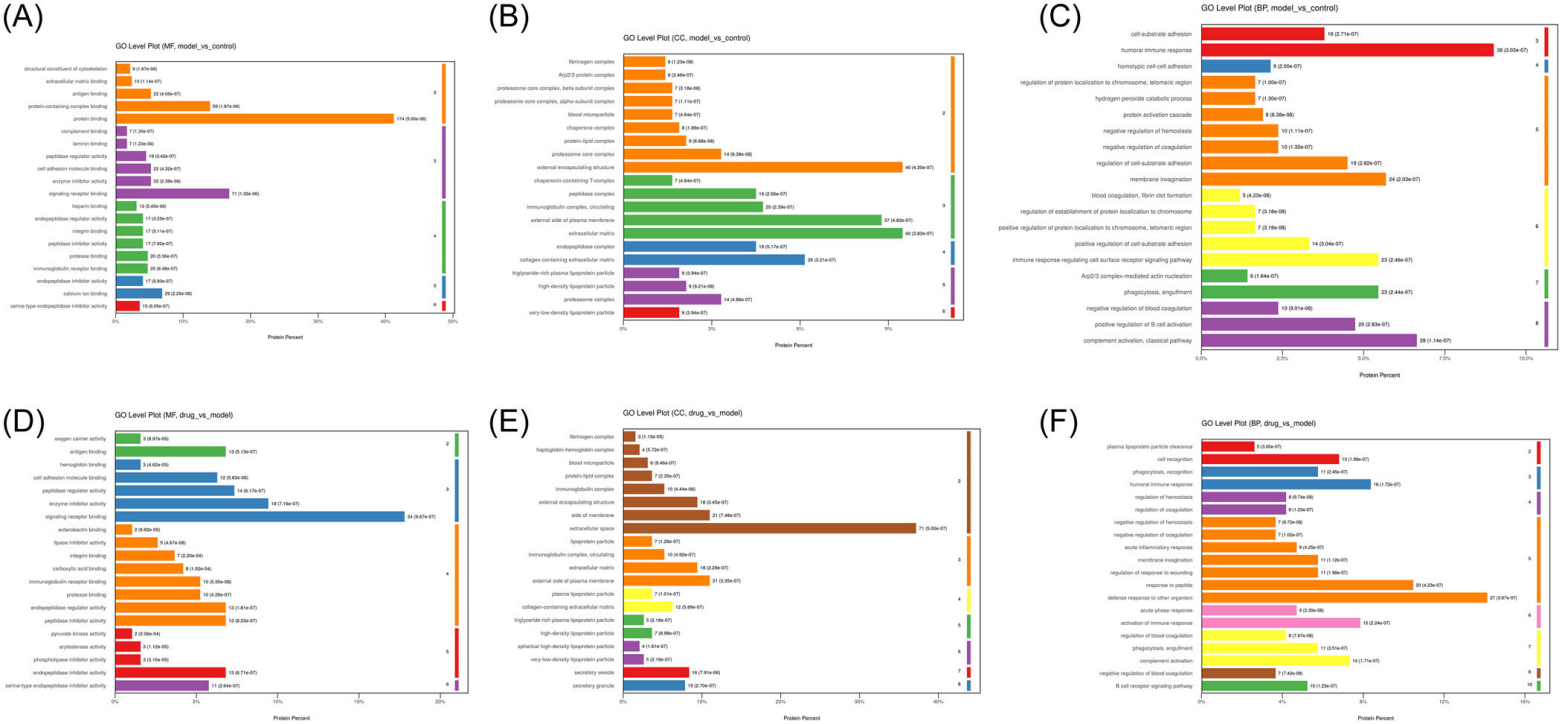
Proteomics analysis of serum exosomes from rats. (A) Molecular function of identified DEPs between CIA and Control groups. (B) Subcellular localization prediction of identified DEPs between CIA and Control groups. (C) The biological process of identified DEPs between CIA and Control groups. (D) Molecular function of identified DEPs between TP and CIA groups. (E) Subcellular localization prediction of identified DEPs between TP and CIA groups. (F) The biological process of identified DEPs between TP and CIA group. CIA, collagen‐induced arthritis; DEPs, differentially expressed proteins; TP, triptolide.

GO enrichment analysis were performed to identify the functions of DEPs. In terms of molecular function, the 422 DEPs between the control and CIA groups were mainly associated with protein binding, signaling receptor binding, protein‐containing complex binding, calcium ion binding, antigen binding, and cell adhesion molecule binding (Figure 4A vs. the Control group). According to the cellular component category, these DEPs primarily participated in the extracellular matrix, external encapsulating structure, external side of the plasma membrane, collagen‐containing extracellular matrix, immunoglobulin complex, circulating and peptidase complex in the CIA group (Figure 4B vs. the Control group). Moreover, the biological process categorization revealed that these DEPs mainly focused on humoral immune response, complement activation, classical pathway, membrane invagination, phagocytosis, engulfment, and immune response‐regulating cell surface receptor signaling pathway (Figure 4C vs. the Control group).

These identified DEPs between the CIA and TP groups were further categorized using GO annotation. In terms of molecular function, the DEPs were mainly associated with signaling receptor binding, enzyme inhibitor activity, peptidase regulator activity, endopeptidase regulator activity, antigen binding, and endopeptidase inhibitor activity in the TP group (Figure 4D vs. the CIA group). According to the cellular component category, these DEPs were chiefly associated with extracellular space, external side of the plasma membrane, side of the membrane, extracellular matrix, external encapsulating structure, and secretory vesicle in the TP group (Figure 4E vs. the CIA group). Moreover, the biological process categorization showed that these DEPs primarily participated in defense response to other organisms, response to peptides, humoral immune response, activation of immune response, complement activation, and cell recognition in the TP group (Figure 4F vs. the CIA group).

### 3.5 Pathway analysis of DEPs in serum exosomes

**Figure 5:**
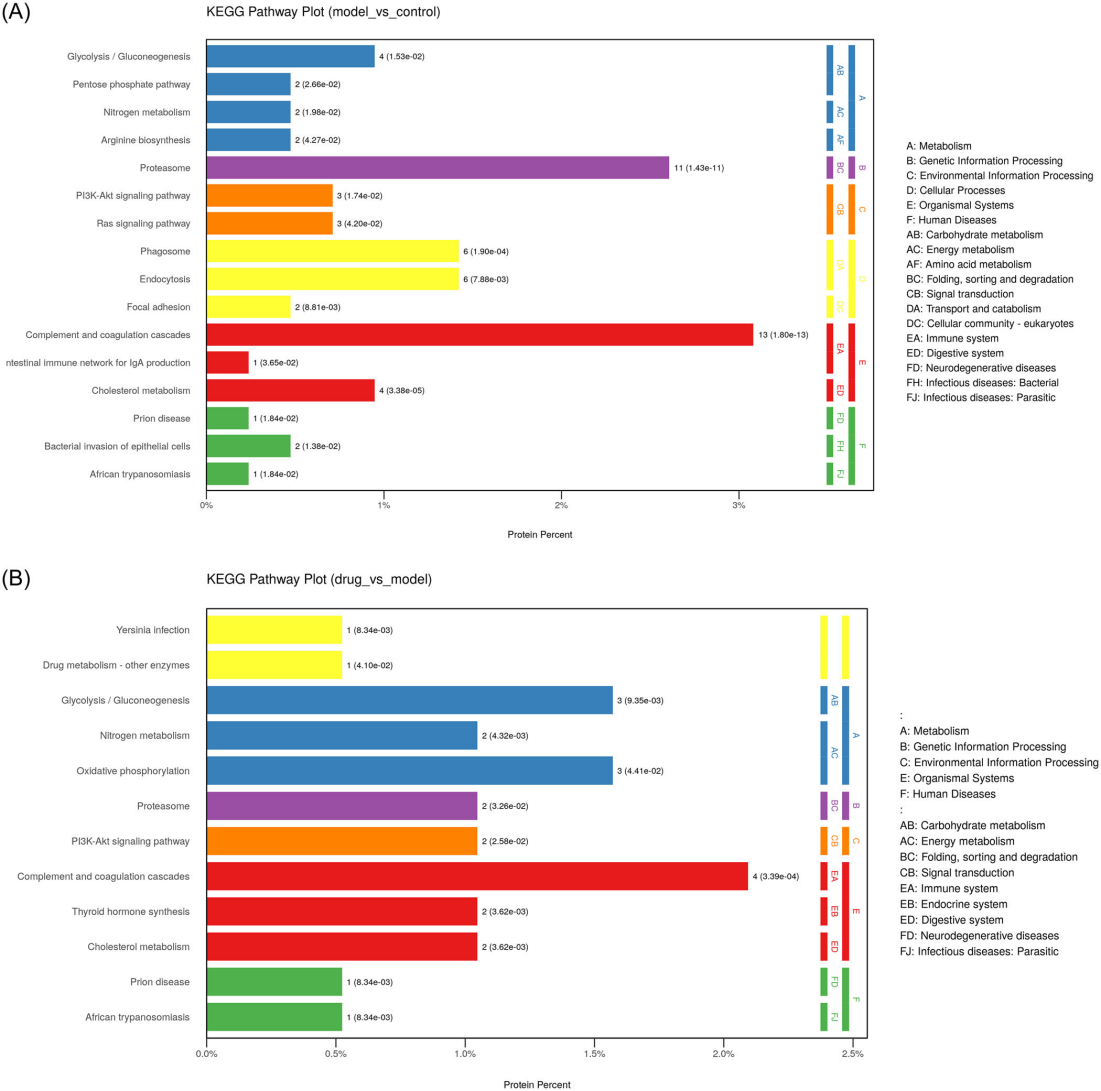
Pathway enrichment analysis of DEPs in serum exosomes. (A) KEGG pathway enrichment analysis of identified DEPs between CIA and Control groups. (B) KEGG pathway enrichment analysis of identified DEPs between TP and CIA groups. CIA, collagen‐induced arthritis; DEPs, differentially expressed proteins; KEGG, Kyoto encyclopedia of genes and genomes; TP, triptolide.

KEGG pathway enrichment analysis demonstrated that 422 DEPs between CIA and Control rats were mapped into 52 pathways, including complement and coagulation cascades, proteasome, cholesterol metabolism, phagosome, endocytosis, and so on. The top 16 most significantly enriched pathways are shown in Figure 5A. Complement and coagulation cascades displayed the greatest number of target connections with 13 targets, including six regulated proteins and seven downregulated proteins. The following enriched pathway Proteasome showed 11 targets, including proteasome 20S subunit (Psm) alpha 1, Psma2, Psma4, Psma6, Psma8, Psmb1, Psmb2, Psmb3, Psmb5, Psmb7, and Proteasome subunit beta, which were downregulated in circulating exosomes from CIA. The third enriched pathway cholesterol metabolism pathway includes apolipoprotein C1 (Apoc1), Apoc2, lipoprotein receptor‐related protein 1 (Lrp1), and Apolipoprotein E (apoE), which were downregulated in circulating exosomes from CIA. In addition, activated pathways in the CIA group include nitrogen metabolism involved in carbonic anhydrase 1 (CA1), carbonic anhydrase 2 (CA2), and IL‐17 signaling pathway (LCN2).

The 191 DEPs between TP and CIA rats were mapped into the 32 pathways, including complement and coagulation cascades, glycolysis/gluconeogenesis, oxidative phosphorylation, cholesterol metabolism, thyroid hormone synthesis, nitrogen metabolism, and so on. The top 16 most significantly enriched pathways are shown in Figure 5B. Complement and coagulation cascades displayed the highest number of target connections with four targets, including alpha‐2‐macroglobulin (A2M), fibrinogen gamma chain (Fgg), complement component 9 (C9), and fibrinogen B beta (Fgb), followed by glycolysis/gluconeogenesis (count = 3).

### 3.6 Screening and validation of proteins responsive to TP treatment

**Figure 6:**
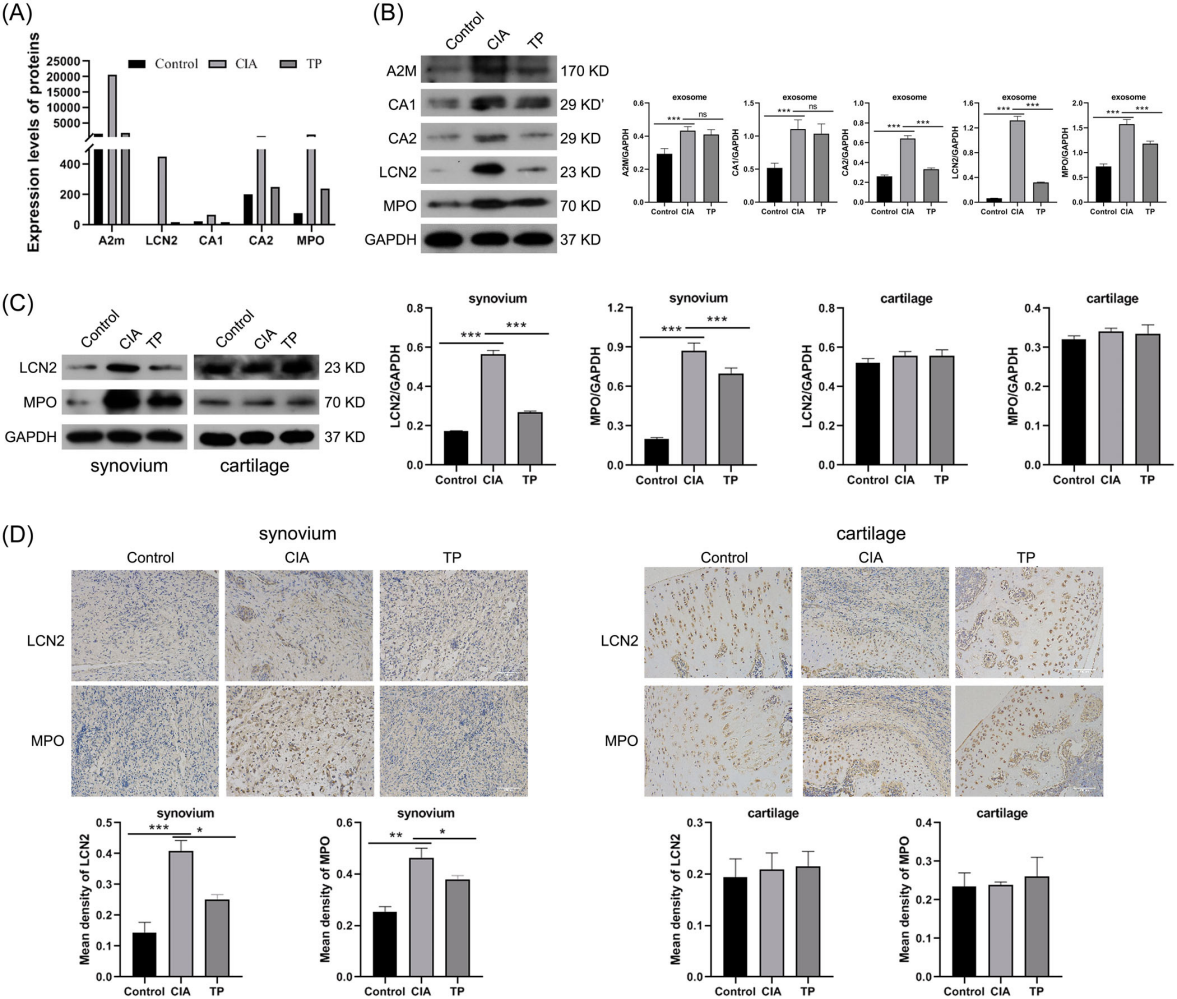
Screening and validation of proteins responsive to TP treatment. (A) Proteomics analysis revealed the expression levels of A2M, LCN2, CA1, CA2, and MPO proteins in serum exosomes from the Control, CIA, and TP rats. (B) Western blot analysis of A2M, CA1, CA2, LCN2, and MPO in serum exosomes from the Control, CIA, and TP rats. (C) Western blot analysis of LCN2 and MPO expression in the cartilage and synovial tissues. (D) Immunohistochemical staining of LCN2 and MPO in the cartilage and synovial tissues. Data are presented as the mean ± SEM (*n* = 6). ns, no significance. *p < .05, **p < .01, ***p < .001. CIA, collagen‐induced arthritis; LCN2, lipocalin 2; MPO, myeloperoxidase; TP, triptolide.

Based on the proteomic profile, we found that A2M was implicated in complement and coagulation cascades, LCN2 was involved in the IL‐17 signaling pathway, CA1 and CA2 were associated with nitrogen metabolism, and the well‐known inflammatory factor myeloperoxidase (MPO) exhibited upregulation in CIA rats which decreased following TP treatment (Figure 6A). Western blot analysis confirmed elevated levels of A2M, CA1, CA2, LCN2, and MPO in exosomes from CIA rats. However, the addition of TP significantly reduced the expression levels of CA2, LCN2, and MPO in rat serum exosomes (Figure 6B), suggesting that TP exerted anti‐inflammatory and therapeutic effects probably by suppressing exosomal LCN2 and MPO.

Furthermore, we measured the expression of LCN2 and MPO in cartilage and synovial tissues using western blot analysis and immunohistochemical staining. The data indicated a significant upregulation of only LCN2 and MPO expression in synovial tissues in the CIA group, which could be partially restored upon TP treatment (Figure 6C). In addition, Immunohistochemical staining corroborated these results (Figure 6D). These data collectively suggested that LCN2 and MPO were the pivotal proteins in mediating the therapeutic effects of TP on CIA.

## 4 DISCUSSION

TP has shown therapeutic potential in various autoimmune diseases, including RA. Previous studies have indicated that TP can modulate the functions of several immune cells such as natural killer cells and T‐helper 17 cells (Th17) cells, while suppressing the production of proinflammatory cytokines and chemokines.^17^, ^18^ Additionally, TP also suppresses cell proliferation and inflammation in fibroblast‐like synoviocytes (FLS).^19^, ^20^ These findings suggest that TP exerts a multi‐target regulatory function. To systematically elucidate the mechanism of TP, we analyzed the impact of TP on circulating exosomal protein expression in CIA model rats using quantitative proteomics. The results revealed that TP treatment significantly alleviated paw swelling and pathological injury of the ankle joint in CIA rats, along with reducing levels of serum inflammatory cytokines. Quantitative proteomic analysis identified significant changes in circulating exosome proteins in CIA rats following TP treatment, particularly decreased expression levels of several proteins enriched in the complement coagulation pathway, IL‐17 signaling pathway, and nitrogen metabolism. Furthermore, we observed that the addition of TP significantly inhibited the levels of LCN2 and MPO in exosomes and synovial tissues, which were increased in CIA rats. Thus, LCN2 and MPO were identified as highly relevant proteins for TP treatment. Collectively, our findings identify a set of exosomal proteins as potential biomarkers for RA treatment while extending the understanding of the disease, providing a scientific basis for unraveling the mechanism underlying TP's efficacy against RA.

Exosomes are a class of extracellular vesicles that are present in various biological fluids.^21^ Exosomes transport multiple signal molecules, including nucleic acids, proteins, lipids, and metabolites, for the exchange of intercellular information and material, thus affecting cellular processes.^22^ The composition of exosome cargos is highly variable depending on the cellular origin and state. Numerous studies have demonstrated that exosomes modulate diverse biological processes and contribute to the pathogenesis of several diseases, including RA.^23^, ^24^ Qin et al.^25^ investigated the exosomal protein profiles in plasma samples from RA patients and controls, identifying 32 upregulated proteins and five downregulated proteins specifically associated with RA and revealed that these exosomes exert immunoregulatory activity and promote the development of RA. Another study identified 28 highly expressed proteins unique to RA uniquely by comparing proteomics data from synovial fluid (SF)‐derived exosomes in RA, axial spondyloarthritis, gout, and osteoarthritis patients.^26^ In our study, we identified 248 upregulated DEPs and 174 downregulated DEPs in CIA rats. Notably, these DEPs were predominantly enriched in complement and coagulation cascades, which are critical for innate responses. Dysregulation or inadvertent activation of the complement and coagulation system contributes to the pathogenesis of autoimmune diseases. Several studies have also reported significant activation of complement and coagulation cascade in RA. Shu et al.^27^ demonstrated that differentially expressed genes in ankle joints of CIA rats were enriched in complement and coagulation cascades. Another study utilizing quantitative proteomics analysis highlighted the involvement of SF‐derived exosome‐specific proteins in complement and coagulation cascades among RA patients.^26^ Collectively, these data suggest that dysregulation of the complement and coagulation cascade plays a pivotal role in the pathogenesis of RA. Additionally, the proteasome pathway emerges as another significantly enriched pathway. Notably, the proteins involved in proteasome, including Psma1, Psma2, Psma4, Psma6, Psma8, Psmb1, Psmb2, Psmb3, Psmb5, Psmb7, and Proteasome subunit beta, were all significantly downregulated expression in CIA. These subunits are integral components of the 26S proteasome complex within the ubiquitin proteasome system‐a primary cellular protein degradation machinery. The proteasome has been recognized as a potential target for mitigating inflammation. Nakamachi et al.^28^ previously reported proteasome subunit Psmb5 was downregulated in RA FLS exosomes, and PSMB5‐silenced exosomes promoted macrophage migration. Another study demonstrated significant downregulation of Psmb1 in the CD4 + T‐cell‐derived exosomes from patients with RA.^29^ Our findings further support these studies by highlighting the potential involvement of circulating exosomes in RA.

Due to the unique function of exosomes in RA, investigating potential drugs that can regulate exosomes has emerged as a novel field of drug research. Natural bioactive products have garnered increasing attention due to their ability to modulate exosome function. Numerous studies have elucidated the therapeutic effects and molecular mechanisms of natural bioactive products on various diseases, particularly focusing on their impact on exosomes.^30^, ^31^, ^32^ Li et al.^7^ revealed that TP promotes chondrocyte proliferation and secretory functions via inhibiting the secretion of exosomal miR‐221 from FLS. Another study showed that TP attenuates the inhibitory effect of ankylosing spondylitis‐derived mesenchymal stem cells on osteoclastogenesis through modulating exosomal transfer of circ_0110634.^33^ In the present study, proteomic analysis and western blot analysis verification identified LCN2 and MPO as the most relevant proteins associated with TP treatment. LCN2, also known as neutrophil gelatinase‐associated lipocalin, is a 25 kDa protein expressed primarily in immune cells, particularly neutrophils. Elevated levels of LCN2 have been reported in both serum and SF samples from RA patients.^34^, ^35^ LCN2 plays an important role in the pathogenesis of RA. Katano et al.^35^ demonstrated that LCN2 released from neutrophils increased the levels of transitional endoplasmic reticulum ATPase, cathepsin D, and transglutaminase 2 proteins in synoviocytes, leading to inhibition of chondrocyte proliferation. Recent experimental studies have demonstrated that LCN2 encapsulated in exosomes acts as a signaling molecule. For example, ischemia‐induced astrocytic exosomal secretion of LCN2 promotes neuronal cell death and neurodegeneration in vitro.^36^ Similarly, increased expression levels of LCN2 in the urinary exosomes were observed in patients with delayed graft function (DGF) compared to non‐DGF patients, indicating its potential as a biomarker for kidney dysfunction in renal transplantation.^37^ In this study, LCN2 was not detected in circulating exosomes from control rats but exhibited high expression in CIA rats. However, TP treatment significantly decreased the expression of LCN2 in exosomes. Furthermore, the expression of LCN2 in the synovial tissues from CIA rats was higher than that in controls. These findings strongly support the hypothesis that the exosomal LCN2 may be involved in the inflammatory response and pathological process in CIA rats and that TP treatment provides protection by preventing these effects mediated by exosomal LCN2. Another protein of interest is MPO, which is mainly derived from neutrophils and can promote inflammatory responses and oxidative stress.^38^ Abundant MPOs have been found in SF and synovial tissues from RA joints and act as mediators of joint inflammation and damage.^39^, ^40^ Moreover, MPO has been identified as a potential predictor of cardiovascular risk among individuals with RA.^41^ In addition, increased levels of MPO encapsulated in exosomes or extracellular vesicles were associated with various diseases, such as type 2 diabetes mellitus and deep venous thrombosis.^42^, ^43^ Consistent with these reports, we also noted significant elevated expression of MPO in exosomes isolated from the serum samples in CIA rats. However, TP treatment led to a significant decrease in exosomal MPO levels. Surprisingly, changes observed regarding MPO expression patterns in the synovial tissues were consistent with those seen within circulating exosomes. Interestingly, LCN2 and MPO are both proteins related to neutrophils which contribute to the initiation and progression of RA. Studies have indicated that neutrophils drive inflammation through various ways, including the release of exosomes.^44^ Jiao et al.^45^ reported that exosomal miR‐30d‐5p derived from neutrophils promotes M1 macrophage polarization and pyroptosis in sepsis‐related acute lung injury. Based on these observations and related studies, we hypothesize that exosomal LCN2 and MPO derived from neutrophils may promote RA joint injury, while TP may alleviate arthritis symptoms of CIA rats by blocking this effect. Indeed, further investigations are warranted to explore whether TP regulates synovial inflammation and joint injury by altering the cargoes of exosomes derived from neutrophils.

Taken together, our results identify a set of exosomal proteins as potential biomarkers for RA treatment and extend our understanding of the disease mechanism. In summary, our study provides novel scientific evidence for elucidating the therapeutic mechanism of TP for RA treatment.

## AUTHOR CONTRIBUTIONS

**Xiuchan Liu**: Conceptualization (lead); investigation (equal); methodology (lead); writing—original draft (lead). **Xu Liu**: Investigation (equal); methodology (lead); formal analysis (lead); visualization (lead); writing—review and editing (equal). **Hui Wang**: Investigation (equal); formal analysis (lead); methodology (equal); visualization (lead); writing—review and editing (lead). **Ming Chen**: Methodology (equal); software (lead); visualization (supporting); writing—review and editing (equal). **Geng Zhang**: Methodology (equal); visualization (supporting); validation (equal); writing—review and editing (equal). **Dongyun Ren**: Methodology (supporting); validation (equal); writing—review and editing (equal). **Na Zhang**: Methodology (supporting); project administration (lead); validation (equal); writing—review and editing (equal). **Wei Wei**: Conceptualization (lead); investigation (lead); project administration (lead); resources; writing—review and editing (lead). All authors agreed to publish this manuscript.

## CONFLICT OF INTEREST STATEMENT

The authors declare no conflict of interest.

## ETHICS STATEMENT

This study was approved by the Ethics Committee of Tianjin Hospital (No. 2023 Medical Ethics Review 069).

## Data Availability

The data sets used and analyzed during the current study are available from the corresponding author upon reasonable request.

## References

[1] Finckh A , Gilbert B , Hodkinson B , et al. Global epidemiology of rheumatoid arthritis. Nat Rev Rheumatol. 2022;18(10):591‐602. 36068354 10.1038/s41584-022-00827-y

[2] Venetsanopoulou AI , Alamanos Y , Voulgari PV , Drosos AA . Epidemiology of rheumatoid arthritis: genetic and environmental influences. Expert Rev Clin Immunol. 2022;18(9):923‐931. 35904251 10.1080/1744666X.2022.2106970

[3] Radu AF , Bungau SG . Nanomedical approaches in the realm of rheumatoid arthritis. Ageing Res Rev. 2023;87:101927. 37031724 10.1016/j.arr.2023.101927

[4] Feng Z , Fu L , Wang J , et al. Efficacy of tripterygium glycosides (TG) in rheumatoid arthritis as a disease‐modifying anti‐rheumatic drug (DMARD) in combination with conventional DMARDs: a systematic review and meta‐analysis of randomized controlled trials. Pharmacol Res. 2022;184:106405. 36028187 10.1016/j.phrs.2022.106405

[5] Luo Y , Hou X , Xi A , Luo M , Wang K , Xu Z . *Tripterygium wilfordii* Hook F combination therapy with methotrexate for rheumatoid arthritis: an updated meta‐analysis. J Ethnopharmacol. 2023;307:116211. 36706936 10.1016/j.jep.2023.116211

[6] Li M , Wang G , Yan Y , et al. Triptolide and l‐ascorbate palmitate co‐loaded micelles for combination therapy of rheumatoid arthritis and side effect attenuation. Drug Delivery. 2022;29(1):2751‐2758. 35999774 10.1080/10717544.2022.2115162 PMC9423844

[7] Li N , Chen Z , Feng W , et al. Triptolide improves chondrocyte proliferation and secretion via down‐regulation of miR‐221 in synovial cell exosomes. Phytomedicine. 2022;107:154479. 36194972 10.1016/j.phymed.2022.154479

[8] Mo C , Zhao J , Liang J , Wang H , Chen Y , Huang G . Exosomes: a novel insight into traditional Chinese medicine. Front Pharmacol. 2022;13:844782. 36105201 10.3389/fphar.2022.844782 PMC9465299

[9] Lu DX , Liu F , Wu H , et al. Wumei pills attenuates 5‐fluorouracil‐induced intestinal mucositis through Toll‐like receptor 4/myeloid differentiation factor 88/nuclear factor‐κB pathway and microbiota regulation. World J Gastroenterol. 2022;28(32):4574‐4599. 36157934 10.3748/wjg.v28.i32.4574 PMC9476879

[10] Li X , Gong S , Chen W , et al. Schisandrol A, a bioactive constituent from Schisandrae Chinensis Fructus, alleviates drug‐induced liver injury by autophagy activation via exosomes. Bioorg Chem. 2023;139:106751. 37531820 10.1016/j.bioorg.2023.106751

[11] Wei Y , Li M , Cui S , et al. Shikonin inhibits the proliferation of human breast cancer cells by reducing tumor‐derived exosomes. Molecules. 2016;21(6):777. 27322220 10.3390/molecules21060777 PMC6274101

[12] Lin W , Shen P , Huang Y , et al. Wutou decoction attenuates the synovial inflammation of collagen‐induced arthritis rats via regulating macrophage M1/M2 type polarization. J Ethnopharmacol. 2023;301:115802. 36209953 10.1016/j.jep.2022.115802

[13] Kong X , Zhang Y , Liu C , et al. Anti‐angiogenic effect of triptolide in rheumatoid arthritis by targeting angiogenic cascade. PLoS One. 2013;8(10):e77513. 24204851 10.1371/journal.pone.0077513 PMC3810371

[14] Yu GM , Zhou LF , Zeng BX , Huang JJ , She X . The antioxidant effect of triptolide contributes to the therapy in a collagen‐induced arthritis rat model. Redox Rep. 2021;26(1):197‐202. 34788192 10.1080/13510002.2021.2004047 PMC8604496

[15] Tai Y , Huang B , Guo P , et al. TNF‐α impairs EP4 signaling through the association of TRAF2‐GRK2 in primary fibroblast‐like synoviocytes. Acta Pharmacol Sin. 2022;43(2):401‐416. 33859345 10.1038/s41401-021-00654-z PMC8791952

[16] Liu S , Fu Y , Mei K , et al. A shedding soluble form of interleukin‐17 receptor D exacerbates collagen‐induced arthritis through facilitating. Cell Mol Immunol. 2021;18(8):1883‐1895. 32963355 10.1038/s41423-020-00548-w PMC8322419

[17] Shen MY , Wang X , Di YX , et al. Triptolide inhibits Th17 differentiation via controlling PKM2‐mediated glycolysis in rheumatoid arthritis. Immunopharmacol Immunotoxicol. 2022;44(6):838‐849. 35657277 10.1080/08923973.2022.2086139

[18] Wang N , Min X , Ma N , et al. The negative impact of triptolide on the immune function of human natural killer cells. Pharmaceuticals. 2023;16(3):458. 36986557 10.3390/ph16030458 PMC10057343

[19] Wen J , Liu J , Wang X , Wang J . Triptolide promotes the apoptosis and attenuates the inflammation of fibroblast‐like synoviocytes in rheumatoid arthritis by down‐regulating lncRNA ENST00000619282. Phytotherapy Res: PTR. 2021;35(8):4334‐4346. 10.1002/ptr.7129 34161642

[20] Wen J , Liu J , Wan L , et al. Triptolide inhibits cell growth and inflammatory response of fibroblast‐like synoviocytes by modulating hsa‐circ‐0003353/microRNA‐31‐5p/CDK1 axis in rheumatoid arthritis. Int Immunopharmacol. 2022;106:108616. 35203042 10.1016/j.intimp.2022.108616

[21] Heydari R , Koohi F , Rasouli M , et al. Exosomes as rheumatoid arthritis diagnostic biomarkers and therapeutic agents. Vaccines. 2023;11(3). 10.3390/vaccines11030687 PMC10057381 36992270

[22] Mahmoudi F , Hanachi P , Montaseri A . Extracellular vesicles of immune cells; immunomodulatory impacts and therapeutic potentials. Clin Immunol (Orlando, Fla.). 2023;248:109237. 10.1016/j.clim.2023.109237 36669608

[23] Sehgal A , Singh S , Sharma N , et al. The emerging role of exosomes in innate immunity, diagnosis and therapy. Inflammopharmacology. 2022;13:1085057. 10.3389/fimmu.2022.1085057 PMC9885214 36726968

[24] Karami Fath M , Azami J , Jaafari N , et al. Exosome application in treatment and diagnosis of B‐cell disorders: leukemias, multiple sclerosis, and arthritis rheumatoid. Cell Mol Biol Letters. 2022;27(1):74. 10.1186/s11658-022-00377-x PMC9446857 36064322

[25] Qin Q , Song R . Systemic proteomic analysis reveals distinct exosomal protein profiles in rheumatoid. Arthritis. 2021;2021:9421720. 10.1155/2021/9421720 PMC8390169 34458379

[26] Huang Y , Liu Y , Huang Q , et al. TMT‐based quantitative proteomics analysis of synovial fluid‐derived exosomes in inflammatory arthritis. Front Immunol. 2022;13:800902. 35359923 10.3389/fimmu.2022.800902 PMC8961740

[27] Shu H , Zhao H , Shi Y , et al. Transcriptomics‐based analysis of the mechanism by which Wang‐Bi capsule alleviates joint destruction in rats with collagen‐induced arthritis. Chin Med. 2021;16(1):31. 33845855 10.1186/s13020-021-00439-w PMC8042720

[28] Nakamachi Y , Uto K , Hayashi S , et al. Exosomes derived from synovial fibroblasts from patients with rheumatoid arthritis promote macrophage migration that can be suppressed by miR‐124‐3p. Heliyon. 2023;9(4):e14986. 37151687 10.1016/j.heliyon.2023.e14986 PMC10161379

[29] Huang L , Liang L , Ji Z , et al. Proteomics profiling of CD4 + T‐cell‐derived exosomes from patients with rheumatoid arthritis. Int Immunopharmacol. 2023;122:110560. 37423153 10.1016/j.intimp.2023.110560

[30] Tang B , Wu Y , Zhang Y , Cheng Y , Wu Y , Fang H . Scorpion and centipede alleviates severe asthma through M2 macrophage‐derived exosomal miR‐30b‐5p. Aging. 2022;14(9):3921‐3940. 35500231 10.18632/aging.204053 PMC9134957

[31] Mo C , Zhao J , Liang J , Wang H , Chen Y , Huang G . Exosomes: a novel insight into traditional Chinese medicine. Front Pharmacol. 2022;13:844782. 36105201 10.3389/fphar.2022.844782 PMC9465299

[32] Chen Z , Wu H , Fan W , et al. Naringenin suppresses BEAS‐2B‐derived extracellular vesicular cargoes disorder caused by cigarette smoke extract thereby inhibiting M1 macrophage polarization. Front Immunol. 2022;13:930476. 35924248 10.3389/fimmu.2022.930476 PMC9342665

[33] Ji W , Lu Y , Ma Z , et al. Triptolide attenuates inhibition of ankylosing spondylitis‐derived mesenchymal stem cells on the osteoclastogenesis through modulating exosomal transfer of circ‐0110634. J Orthopaedic Translation. 2022;36:132‐144. 10.1016/j.jot.2022.05.007 PMC9489540 36185580

[34] Gulkesen A , Akgol G , Poyraz AK , et al. Lipocalin 2 as a clinical significance in rheumatoid arthritis. Cent Eur J Immunol. 2017;3(3):269‐273. 10.5114/ceji.2017.70969 PMC5708208 29204091

[35] Katano M , Okamoto K , Arito M , et al. Implication of granulocyte‐macrophage colony‐stimulating factor induced neutrophil gelatinase‐associated lipocalin in pathogenesis of rheumatoid arthritis revealed by proteome analysis. Arthritis Res Ther. 2009;11(1):3. 10.1186/ar2587 PMC2688233 20527084

[36] Liu R , Wang J , Chen Y , et al. NOX activation in reactive astrocytes regulates astrocytic LCN2 expression and neurodegeneration. 2022;13(4):371. 10.1038/s41419-022-04831-8 PMC9018876 35440572

[37] Alvarez S , Suazo C , Boltansky A , et al. Urinary exosomes as a source of kidney dysfunction biomarker in renal transplantation. Transplant Proc. 2013;45(10):3719‐3723. 24315007 10.1016/j.transproceed.2013.08.079

[38] Tucker B , Ephraums J , King TW , Abburi K , Rye KA , Cochran BJ . Impact of impaired cholesterol homeostasis on neutrophils in Atherosclerosis. Arterioscler Thromb Vasc Biol. 2023;43(5):618‐627. 36951066 10.1161/ATVBAHA.123.316246

[39] Birkelund S , Bennike TB , Kastaniegaard K , et al. Proteomic analysis of synovial fluid from rheumatic arthritis and spondyloarthritis patients. Clin Proteomics. 2020;17:29. 32782445 10.1186/s12014-020-09292-9 PMC7412817

[40] Umar S , Umar K , Sarwar AHMG , et al. *Boswellia serrata* extract attenuates inflammatory mediators and oxidative stress in collagen induced arthritis. Phytomedicine. 2014;21(6):847‐856. 24667331 10.1016/j.phymed.2014.02.001

[41] Montes EG , Mansani FP , de Oliveira Toledo Júnior A , et al. Myeloperoxidase as an important predictor of cardiovascular risk in individuals with rheumatoid arthritis. Inflammopharmacology. 2021;29(6):1819‐1827. 34825303 10.1007/s10787-021-00892-x

[42] Peng L , Li X , Li Y , et al. Increased concentrations of myeloperoxidase in serum and serum extracellular vesicles are associated with type 2 diabetes mellitus. Clin Chim Acta. 2021;522:70‐76. 34390687 10.1016/j.cca.2021.08.010

[43] Han Y , Bai X , Wang X . Exosomal myeloperoxidase as a biomarker of deep venous thrombosis. Ann Transl Med. 2022;10(1):9. 35242854 10.21037/atm-21-5583 PMC8825553

[44] Wright HL , Lyon M , Chapman EA , Moots RJ , Edwards SW . Rheumatoid arthritis synovial fluid neutrophils drive inflammation through production of chemokines, reactive oxygen species, and neutrophil extracellular traps. Front Immunol. 2020;11:584116. 33469455 10.3389/fimmu.2020.584116 PMC7813679

[45] Jiao Y , Zhang T , Zhang C , et al. Exosomal miR‐30d‐5p of neutrophils induces M1 macrophage polarization and primes macrophage pyroptosis in sepsis‐related acute lung injury. Crit Care. 2021;25(1):356. 34641966 10.1186/s13054-021-03775-3 PMC8507252

